# A single center’s experience of the extrapulmonary neuroendocrine carcinomas

**DOI:** 10.14744/nci.2021.47887

**Published:** 2021-02-14

**Authors:** Emir Celik, Nilay Sengul Samanci, Sumeyra Derin, Sahin Bedir, Ezgi Degerli, Kerem Oruc, Nihan Senturk Oztas, Gulin Alkan, Abdulhalim Senyigit, Zeynep Hande Turna

**Keywords:** Extrapulmonary neuroendocrine carcinoma, liver metastasis, platinum and etoposide

## Abstract

**Objective:**

Extrapulmonary neuroendocrine carcinoma (EP-NEC) is a rare tumor type, and a standard therapy for EP-NEC has not yet been established. The purpose of this research was to explore the overall survival (OS) and therapeutic effects of platinum-etoposide combination therapy in EP-NEC.

**Methods:**

This retrospective study was conducted based on the medical records from January 2010 to March 2020. Eligible patients had been pathologically diagnosed with EP-NEC.

**Results:**

Forty-seven patients were included in the study. About 72.3% (n=34) of the patients were diagnosed with metastatic disease at the first diagnosis. The most common primary tumor site was the stomach. The median progression-free survival (PFS) of the patient group, who received the combination of platinum/etoposide, was 5.83 months (95% CI 4.46–7.20), whereas the median OS of the patients, who were found to have metastatic disease at the first diagnosis, was 13.6 months (95% CI 9.01–18.18). There was no difference in PFS and OS between patients with and without liver metastasis.

**Conclusion:**

The outcome of advanced EP-NECs with platinum/etoposide chemotherapy remains poor. Obviously, there is a need for new, more effective treatment options.

**N**euroendocrine neoplasms can be diagnosed as either well-differentiated tumors or poorly differentiated carcinomas according to their morphology and proliferation indexes (Ki-67 and mitotic index) [1]. The most common site of the primary tumor is small intestine (30.4%) and lung (29.8%), respectively [2]. Neuroendocrine neoplasms are categorized into three groups according to their grade designated by the World Health Organization classification [3]. The ones with a mitotic count of <2/10 high-power fields (HPF) and/or with a Ki-67 index of ≤2% are classified as the neuroendocrine tumor (NET) Grade I, whereas those with a mitotic count of 2/10 to 10/10 HPF and/or with a Ki 67 index between 3% and 20% are classified as NET Grade II. The neuroendocrine carcinoma (NEC), which is grade III, has a mitotic count of more than 20/10 HPF and/or a Ki 67 index >20% [3].

High-grade NECs are aggressive tumors, the vast majority of which originate from the lung, and are characterized by very fast growing cells [4]. On the other hand, in the extrapulmonary NEC (EP-NEC) cases, the primary tumor is most often in the gastrointestinal (GI) tract [4]. Despite the fact that the diagnosis and treatment approaches for high-grade lung NEC (including small cell and large cell carcinomas) have become more prominent, data on the EP-NEC are still limited. Surgical resection, which has a curative potential, cannot be performed in most of the EP-NEC cases, due to the rate of metastatic disease in the initial diagnosis of up to 85% [5]. Current, while chemoradiotherapy is used for limited stage lung NEC, systemic platinum-based chemotherapy is preferred in patients with extensive stage disease [5].

Platinum/etoposide combination chemotherapy has been used as the first-line treatment since the 1990s, due to the histological similarity with small cell lung cancer and the presence of anti-tumor activity [6, 7]. Median overall survival (OS) without chemotherapy can be as short as 1 month [6, 8]. The median progression-free survival (PFS) and the median OS reported in a European multicenter study, in which 113 NEC cases of GI origin were analyzed by Heetfeld et al. in 2015, were 5 months and 16.4 months, respectively; compared to the median PFS and the median OS reported in another multicenter study conducted in Japan on a series of 46 NEC cases of GI origin treated with platinum/etoposide combination, which were 4.0 months and 7.3 months, respectively [9, 10].

We herein studied the clinicopathological profile and the treatment outcome of patients with local/regional and metastatic EP-NECs from a single institution in Turkey.

## Patients and Methods

We retrospectively evaluated the medical records of patients that were histopathologically diagnosed with high-grade EP-NEC and who were treated with combination etoposide and platinum (cisplatine or carboplatine) as the first-line chemotherapy. In addition to the patients, who were found to have metastatic disease at the first diagnosis, patients with local/regional disease were also included in the study. On the other hand, patients with mixed adenoneuroendocrine carcinoma histology were excluded from the study. Patients with lung lesions were also excluded from the study as no distinction could be made between primary and metastases.

In patients with metastatic disease, PFS was defined as the time from the 1st day of starting platinum/etoposide combination therapy to the time of disease progression. In patients with local/regional disease, relapse-free survival (RFS) was defined as the time from the 1st day of starting chemotherapy to the time of disease recurrence. OS was defined as the time from cancer diagnosis to the time of death. The general conditions of patients were assessed by Eastern Cooperative Oncology Group Performance Status (ECOG-PS). Continuous variables are expressed as means or medians according to the normality assumption. Frequencies and proportions were used for categorical variables. The median OS, PFS, and RFS were calculated using the Kaplan–Meier method.

**Highlight key points**

• The first two primary tumor sites were determined as stomach (27.6%) and unknown primary (23.4%). At the time of initial diagnosis, 72.3% of the patients were diagnosed with metastatic cancer.
• Liver metastasis was detected in 44.6% of patients, and none of the patients was found to have cranial metastasis at the first diagnosis.
• Progression-free survival for patients with metastatic disease was 5.83 months (95% CI 4.46–7.20), and the median overall survival was 13.6 months (95% CI 9.01–18.18).
• None of local/regional extrapulmonary neuroendocrine carcinomas cases were administered prophylactic cranial irradiation. Only one patient developed cranial metastasis during the follow-up period.

All statistical analyzes were performed using SPSS Statistics for Windows, version 21.0 (SPSS Inc., Chicago, Ill., USA). Ethics Committee Approval: Cerrahpaşa Clinical Research Ethics Committee granted approval for this study (date: 09.09.2020 number:117340).

## Results

### Patient Characteristics

**Figure 1.**
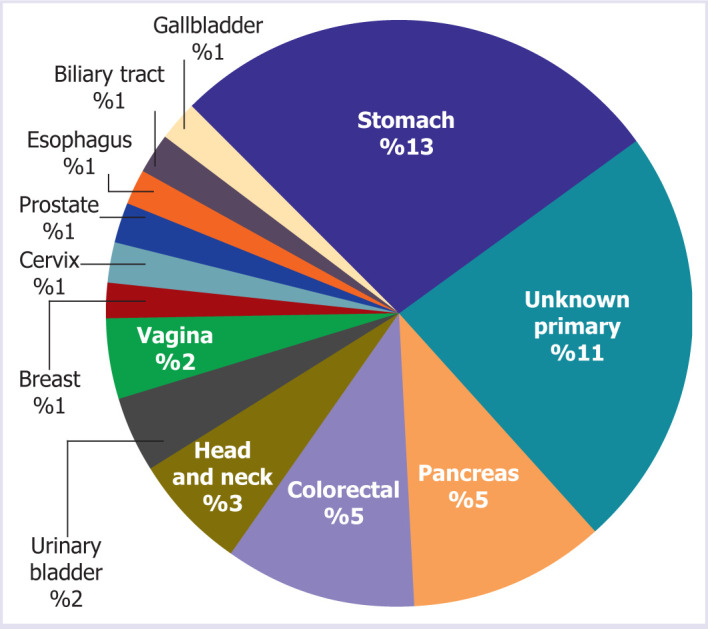
Location of the primary tumor in all cases (local/regional and metastatic disease).

We have identified 47 patients, who were followed up in our center between January 2010 and March 2020 with the histopathological diagnosis of EP-NEC. Demographics and clinical characteristics of these patients are shown in Table 1. In parallel with the literature, most of the patients were male and under the age of 65 [4, 11]. About 72.3% (n=34) of the patients were diagnosed with metastatic disease at the first diagnosis, whereas 27.7% (n=13) of the patients were diagnosed with local/regional disease. The female/male patient ratio was 17/30. Most of the patients were <65 years old and their ECOG-PS was 0–1 except two of the patients. The sites of the primary tumor for all cases are shown in Figure 1. The first five primary tumor sites were determined as stomach (n=13), unknown primary (n=11), pancreas (n=5), colorectal (n=5), and head and neck (n=3), respectively. In 55.3% of cases, the primary tumor site (n=26) was GI tract. None of the patients was found to have cranial metastasis at the first diagnosis. Median Ki-67 index was 80 (range 60–100). Most cases were presenting the histology of small cell carcinoma (Table 1).

**Table 1.** Baseline clinic and demographic characteristics of 47 patients with high-grade extrapulmonary neuroendocrine carcinoma

Variable	(%)
Age	
>65 years	38.3
<65 years	61.7
ECOG PS	
0–1	95.7
2	4.3
Sex	
Female	36.2
Male	63.8
Smoking	
Yes	31.9
No	23.4
Unknown	44.7
Stage at first diagnosis	
Local/regional	27.7
Metastatic	72.3
Initial brain metastasis	0
Ki-67	
>80	80.8
60–80	6.4
Unknown	12.8
Histology	
Small cell	61.7
Large cell	8.5
Unknown	29.8
CT type (for metastatic disease)	
Cisplatin+etoposide	82.3
Carboplatin+etoposide	11.8
No treatment	5.9

ECOG PS: Eastern cooperative oncology group performance status; CT: Chemotherapy.

### Clinical Outcomes

**Figure 2.**
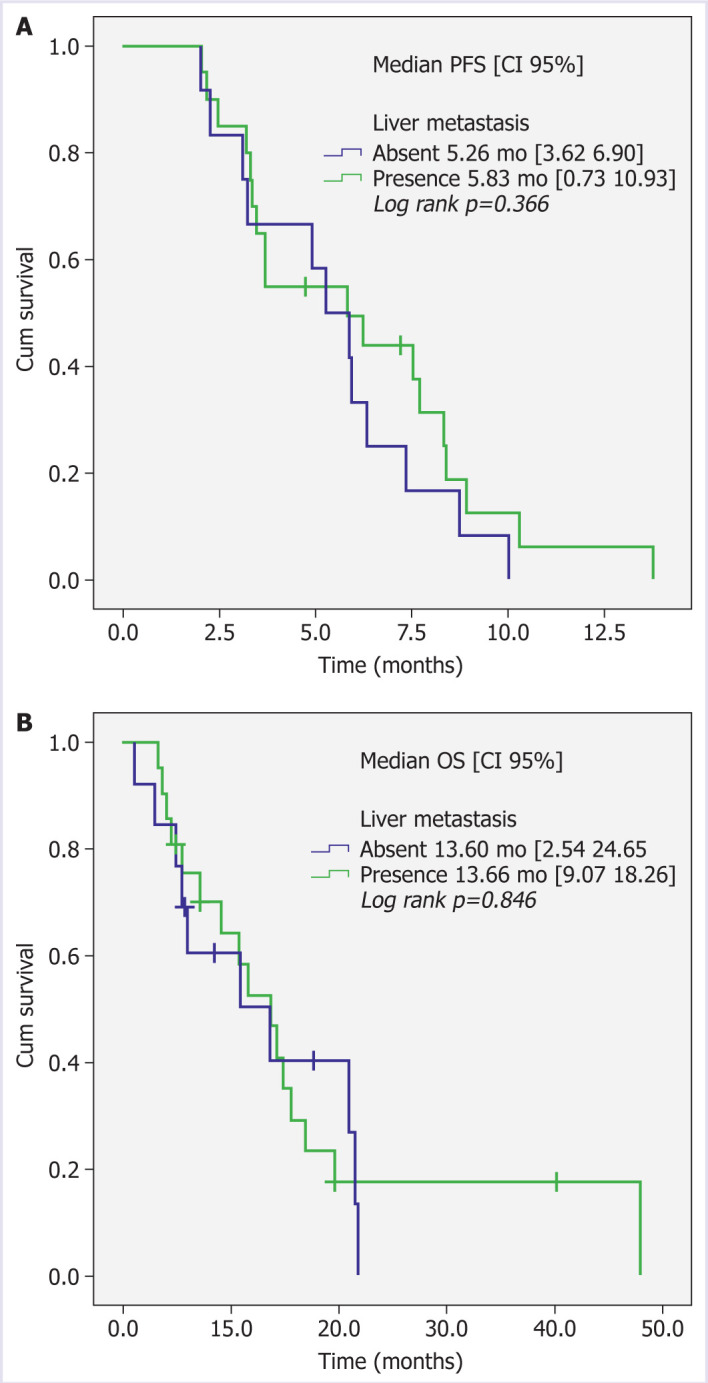
Progression-free survival **(A)** and overall survival **(B)** for patients with the liver metastases.

Of the 34 patients, who were found to have metastatic disease at the first diagnosis, 28 patients received cisplatin/etoposide chemotherapy and four patients received carboplatin/etoposide chemotherapy as the first-line treatment regimens. The remaining two patients died before any treatment could be administered after the diagnosis. Therefore, the PFS analysis was performed with 32 patients and the OS analysis was performed with 34 patients. The median PFS of the patient group, who received the combination of platinum/etoposide, was 5.83 months (95% CI 4.46–7.20), whereas the median OS of the patients, who were found to have metastatic disease at the first diagnosis, was 13.6 months (95% CI 9.01–18.18). Liver metastasis was detected in 21 of 34 patients, who were found to have metastatic disease at the first diagnosis. Sites of metastases other than the liver were lymph node, bone, soft tissue, and skin. Median PFS values of patients with and without liver metastasis were found to be 5.83 months (95% CI 0.73–10.93) and 5.26 months (95% CI 3.62–6.90), respectively (log rank p=0.366) (Fig. 2a); whereas the median OS of patients with and without liver metastasis was found to be 13.66 months (95% CI 9.07–18.26) and 13.6 months (95% CI 2.54–24.65), respectively (log rank p=0.846) (Fig. 2b). Of the 30 patients, who had progressive disease and received first-line chemotherapy, 19 were able to receive second-line chemotherapy.

**Figure 3.**
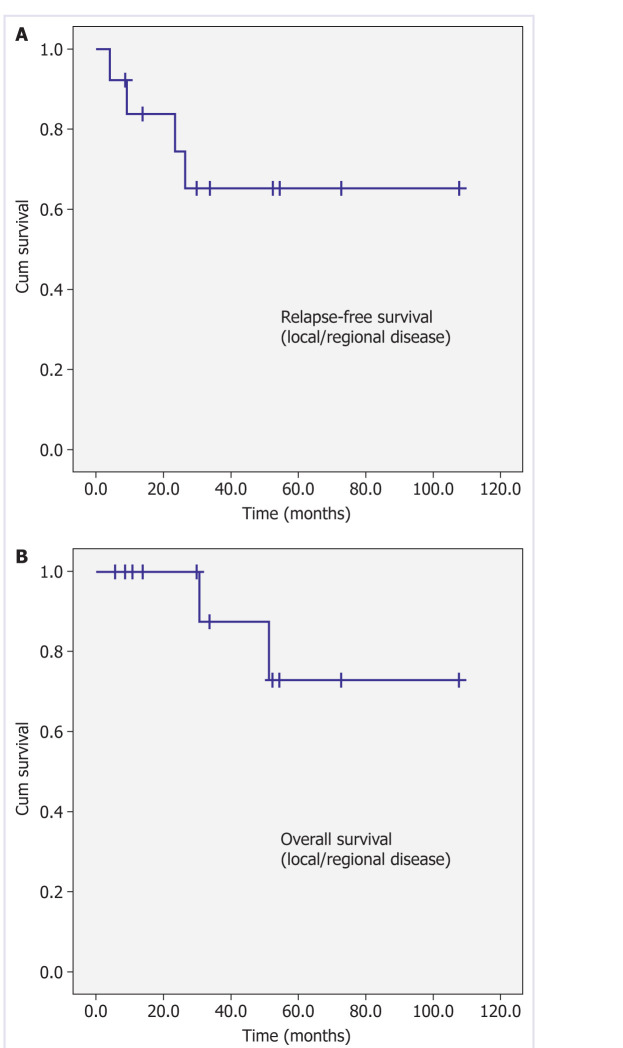
Relapse-free survival **(A)** and overall survival **(B)** for patients with local/regional disease.

The number of cases, who were found to have local/regional disease at the first diagnosis, was 13. Primary tumor site in these patients was GI tract (n=6), head and neck (n=3), unknown primary (n=3), and vagina (n=1). The median follow-up period of these patients was 33.7 months (range 5.6–107.8). Recurrence developed in four patients during the follow-up period, and two patients had died. RFS and OS are shown in Figure 3. However, the median values could not be achieved as there were not enough events during the follow-up period. Six patients, who had stomach (n=3), colorectal (n=2), and gall-bladder (n=1) NEC diagnosis, with the primary lesions in GI tract, underwent surgical procedure and were administered adjuvant chemotherapy. Five of the seven patients, with primary lesions in sites other than GI tract, were administered chemotherapy and radiotherapy, and two patients received only chemotherapy. None of the 13 EP-NEC cases, who were found to have local/regional disease at the first diagnosis, were administered prophylactic cranial irradiation (PCI) in addition to the treatment of the primary lesion. Only one patient developed cranial metastasis during the follow-up period. Disease has recurred with brain metastasis in a patient with vaginal NEC approximately 2 years after the completion of initial chemotherapy and primary radiotherapy.

## Discussion

In this study, the clinical course of the patient population diagnosed with EP-NEC consisting of both local/regional and metastatic patients, who were followed up in a single center, was reported. The group of EP-NEC patients was highly heterogeneous and since the incidence of EP-NEC is also rarer than lung NEC, it could not yet been validated which treatment regimen is more effective along with chemotherapy through a randomized controlled trial with a large number of patients. Most of the trials reported in the literature are retrospective studies and they include a limited number of centers and patients [11–13].

The Surveillance, Epidemiology, and End Results (SEER) program published in 2018 on the NEC epidemiology has revealed very important data [4]. About 8.7% (n=14,732) of the 162,983 NEC cases diagnosed between 1973 and 2012 were EP-NECs. The primary tumor was in GI tract in approximately 37% (n=5,509) of these patients, whereas 28% (n=4,151) of the patients had unknown primary tumor, 34% (n=5,072) of the patients had the primary tumor in other sites. Unfortunately, the relationship between the therapy and survival could not be assessed due to the lack of systemic chemotherapy data used in the SEER study [4]. In comparison, in our study, primary tumor was in GI tract in 55.3% of patients, whereas 23.4% of the patients had unknown primary tumor, and 23.2% of the patients had the primary tumor in other sites. In neuroendocrine neoplasm patients with unknown primary tumor, the site is most frequently localized in the intestine or the lung [14]. Due to the fact that GI tract was determined as the most common site for EP-NEC in both in our study and in other studies reported in the literature, it is important to evaluate biopsies to be taken from these regions in terms of neuroendocrine neoplasms and correct histopathological classification is also vital in terms of the choice of treatment options.

One of the important studies in this line of research is the NORDIC study, in which the treatment responses and prognoses of 305 patients with GI NEC were reported [8]. About 82.6% of the patients included in this study received chemotherapy, whereas 17.4% of them were followed up by only supportive care. The median OS of the patients, who did and who did not receive chemotherapy, was 1 month (95% CI 0.3–1.8) and 11 months (95% CI 9.4–12.6), respectively. The median PFS of the patients, who received chemotherapy, was 4 months (95% CI 3.4–4.6). There was no significant PFS difference between the patients that received cisplatin/etoposide chemotherapy and carboplatin/etoposide chemotherapy [8]. In a prospective cohort study on poorly differentiated NEC in GI tract, PFS and OS of 152 patients treated with platinum/etoposide chemotherapy were reported as 6.2 months and 11.6 months, respectively [15]. In comparison, in our study, following the platinum/etoposide chemotherapy, we have found the median PFS, in patients diagnosed with metastatic EP NEC, as 5.83 months (95% CI 4.46–7.20), and the median OS, in patients with metastatic diagnosis at first diagnosis, as 13.6 months (95% CI 9.01–18.18). Comparison with cisplatin could not be made because the number of patients that received carboplatin was not sufficient. Another finding of our study was that liver metastasis did not have any effect on PFS and OS. However, this result needs to be confirmed by other studies to be conducted with more number of patients, due to the low number of cases in our study. Another contribution of our study to the literature, although it was conducted on few patients, is that it has provided information about the prognosis of patients in the local/regional stage. The median follow-up period in the group of 13 patients with local/regional disease was found to be 33.7 months (range 5.6–107.8 months), whereas four patients developed recurrence and two patients died during the follow-up. Median PFS and OS have not been reached yet as there were not enough events. In fact, the prognosis for local/regional disease was better than expected for this disease group, which is characterized by very fast growing cells in the metastatic stage and short survival times. The curative treatment with the highest curative potential should be determined by combining surgery, radiotherapy, and chemotherapy modalities. Unfortunately, the data in this field have been limited to retrospectively collect case reports and case series.

In small-cell lung cancer, the 2-year cumulative risk of developing brain metastasis is more than 50% and the median survival time after brain metastasis is only 4–5 months [16]. Approximately 65% of patients have brain metastasis detectable on autopsy [17]. Therefore, PCI in both limited and extensive stages in small-cell lung cancer positively affects survival [16]. However, the survival benefit of PCI on EP-NEC has not been clearly demonstrated [18] and is therefore not routinely implemented. In our study, no patient had brain metastasis at the first diagnosis. None of the 13 patients with local/regional disease underwent PCI following the treatment of the primary lesion, and only in one of the patient’s brain metastasis has developed during the follow-up period. As a result, there is still not enough data to support the routine administration of PCI in EP-NEC cases.

### Conclusion

Progress has been made in the treatment of NETs, but the prognosis of high-grade metastatic EP-NEC remains dismal.
